# Pediatric, adult, and late onset multiple sclerosis: Cognitive phenotypes and gray matter atrophy

**DOI:** 10.1002/acn3.52291

**Published:** 2025-01-24

**Authors:** Ermelinda De Meo, Emilio Portaccio, Rosa Cortese, Luis Ruano, Benedetta Goretti, Claudia Niccolai, Francesco Patti, Clara Chisari, Paolo Gallo, Paola Grossi, Angelo Ghezzi, Marco Roscio, Flavia Mattioli, Chiara Stampatori, Marta Simone, Rosa Gemma Viterbo, Raffaello Bonacchi, Assunta Maria Rocca, Elisa Leveraro, Antonio Giorgio, Nicola De Stefano, Massimo Filippi, Matilde Inglese, Maria Pia Amato

**Affiliations:** ^1^ Department of Neuroinflammation, Institute of Neurology Univeristy College of London London UK; ^2^ NEUROFARBA Department, Neurosciences Section University of Florence Florence Italy; ^3^ Azienda Ospedaliera Universitaria Careggi Florence Italy; ^4^ Department of Medicine, Surgery and Neuroscience University of Siena Siena Italy; ^5^ EPIUnit, Instituto de Saúde Pública de Universidade do Porto Porto Portugal; ^6^ Neurology Department Centro Hospitalar de Entre Douro e Vouga Santa Maria da Feira Portugal; ^7^ IRCCS Fondazione Don Carlo Gnocchi Florence Italy; ^8^ University of Catania Catania Italy; ^9^ University of Padova Padova Italy; ^10^ Department, ASST Crema, Neuroimmunology Center, Cardiocerebrovascular Crema Italy; ^11^ Gallarate Hospital Varese Italy; ^12^ ASST Spedali Civili Brescia Neuropsychology Unit Brescia Italy; ^13^ Department of Basic Medical Sciences, Child and Adolescence Neuropsychiatry Unit Neuroscience and Sense Organs University “Aldo Moro” Bari Bari Italy; ^14^ Neuroradiology Unit IRCCS San Raffaele Scientific Institute Milan Italy; ^15^ Neuroimaging Research Unit IRCCS San Raffaele Scientific Institute Milan; ^16^ Department of Neurology, Rehabilitation, Ophthalmology, Genetics, Maternal and Child Health University of Genoa Genoa Italy; ^17^ Neurology Unit IRCCS San Raffaele Scientific Institute Milan Italy; ^18^ IRCCS Ospedale Policlinico San Martino Genoa Italy

## Abstract

**Objectives:**

We aim to investigate cognitive phenotype distribution and MRI correlates across pediatric‐, elderly‐, and adult‐onset MS patients as a function of disease duration.

**Methods:**

In this cross‐sectional study, we enrolled 1262 MS patients and 238 healthy controls, with neurological and cognitive assessments. A subset of 222 MS patients and 92 controls underwent 3T‐MRI scan for brain atrophy and lesion analysis. Multinomial probabilistic models identified likelihood of belonging to cognitive phenotypes (“preserved‐cognition,” “mild verbal memory/semantic fluency,” “mild multi‐domain,” “severe attention/executive,” and “severe multi‐domain”) and experiencing MRI abnormalities based on disease duration and age at onset.

**Results:**

In all groups, the likelihood of “preserved‐cognition” phenotype decreased, whereas “mild multi‐domain” increased with longer disease duration. In pediatric‐ and adult‐onset patients, the likelihood of “mild verbal memory/semantic fluency” phenotypes decreased with longer disease duration, and that of “severe multi‐domain” increased with longer disease duration. Only in adult‐onset patients, the likelihood of “severe executive/attention” phenotype increased with longer disease duration. All groups displayed escalating probabilities of cortical, thalamic, hippocampal, and deep gray matter atrophy over disease course. Compared to adult, pediatric‐onset patients showed lower probability of experiencing thalamic atrophy with longer disease duration, while elderly‐onset showed higher probability of experiencing cortical and hippocampal atrophy.

**Interpretation:**

Age at MS onset significantly influences the distribution of cognitive phenotypes and the patterns of regional gray matter atrophy throughout the disease course.

## Introduction

The onset of multiple sclerosis (MS) usually occurs between the ages of 18 and 50 years, which we define as adult‐onset MS (AOMS). However, up to 10% of patients experience their first attack before age 18 years (commonly termed pediatric‐onset MS; POMS), whereas between 0.6% and 12% of patients present their first symptoms after age 50 (commonly termed elderly‐onset MS; EOMS).^1^, ^2^

Specific features have been identified for both POMS and EOMS, highlighting the significant role of age at disease onset in shaping MS‐related clinical features. While patients with POMS more commonly follow a relapsing–remitting course of the disease,^3^ those with EOMS show a higher incidence of progressive phenotypes.^4^ In POMS, a longer time to reach more severe clinical disability has been reported,^5^ whereas EOMS is characterized by a faster progression of clinical disability.^6^

Cognitive impairment is a relevant feature of both POMS and EOMS. The onset of MS during brain development likely leads to a distinctive pattern of cognitive deficits in POMS, predominantly influenced by the maturation status of various cognitive domains: domains that are immature at the time of MS onset are likely to be more severely affected than those that are already mature.^7^, ^8^ Indeed, POMS patients more frequently exhibit deficits in linguistic abilities and complex attention compared to AOMS.^7^ These cognitive functions rely on brain regions that mature later during development, in late childhood and adolescence.^9^

Similarly, the interplay between brain aging and MS onset could explain the different patterns of cognitive impairment observed in EOMS compared to AOMS.^10^ Specifically, EOMS patients exhibit significantly greater impairment in tasks involving visual learning, memory, and working memory,^10^ cognitive functions that are particularly susceptible to age‐related decline.

Overall, while some differences in cognitive functioning between AOMS, POMS, and EOMS have been identified, the conventional dichotomous classification as either “preserved” or “impaired” may have hampered the identification of specific cognitive profiles prevalent among MS patients based on the age at disease onset. In this context, our novel classification of cognitive functioning into “cognitive phenotypes” (“preserved‐cognition,” “mild verbal memory/semantic fluency,” “mild multi‐domain,” “severe‐attention/executive,” and “severe‐multi‐domain”) may better facilitate achieving this objective.^11^, ^12^, ^13^

Hypothesizing a different prevalence of the cognitive phenotypes according to the age at disease onset and considering the different resilience of developing and aging brain against MS‐related damage, in this cross‐sectional study we aimed to assess and compare (1) the distribution of our newly defined cognitive phenotypes^11^ in MS patients grouped by age at disease onset (EOMS, AOMS, and POMS); (2) the prevalence of each phenotype within each group according to disease duration; and (3) the MRI abnormalities underlying the differences in distribution and prevalence of cognitive phenotypes over the disease course among the MS patients grouped as above.

## Methods

### Ethics committee approval

Approval was received from the local ethical standards committees on human experimentation, and written informed consent was obtained from all participants prior to study enrolment.

### Study subjects

In this cross‐sectional study, we expanded the cohort of our original study (1212 MS patients and 196 HC)^11^ between January 2021 and October 2022, by enrolling additional 50 adult patients with MS,^14^ and 42 sex‐, age‐, and education‐matched healthy controls (HC) with no previous history of neurological dysfunction, from nine Italian MS Centers. Exclusion criteria for all subjects were history of neurological/medical disorders (other than MS for patients), use of antidepressants or other psychoactive drugs, history of learning disability, severe head trauma, alcohol or drug abuse. Other exclusion criteria specific for MS patients were the presence of relapses or corticosteroid use within 4 weeks preceding neuropsychological assessment.^15^ From the whole cohort, we identified three groups according to age at disease onset (based on symptom onset): POMS (age at disease onset <18), AOMS (age at disease onset between 18 and 50), and EOMS (age at disease onset >50).

### Neuropsychological evaluation

Following the original study protocol, all subjects enrolled underwent neuropsychological evaluation including the Rao's Brief Repeatable Battery^12^ and Stroop Coluor and Word Test.^13^ The Rao's Brief Repeatable Battery assesses the most frequently impaired cognitive ldomains in MS, incorporating tests of verbal learning and memory (Selective Reminding Test including long‐term storage, consistent long‐term retrieval and delayed recall); visual/spatial learning and memory (10/36 Spatial Recall Test and its delayed recall); complex attention and information processing speed (Paced Auditory Serial Addition Test and Symbol Digit Modalities Test; and verbal fluency on semantic stimulus Word List Generation). The Stroop Colour and Word Test^13^ assesses complex attention and aspects of executive functioning such as the ability to inhibit cognitive interference.

Corrected scores for age, sex, and education according to normative values^16^ were obtained for each test. To standardize the individual corrected scores, z‐scores for each cognitive test were calculated based on the HC enrolled.

### Neurological assessment

On the day of neuropsychological evaluation, all patients underwent a neurological examination with a rating of the Expanded Disability Status Scale (EDSS) score^17^ and the definition of clinical phenotype.

### MRI data acquisition

Three of nine involved centers (San Raffaele Hospital in Milan, Quantitative Neuroimaging Laboratory of the University of Siena, and Ospedale Policlinico San Martino, Genoa) also performed MRI examination at the time of neuropsychological evaluation, comprising a total of 222 MS patients and 92 HC.

A 3.0 Tesla Philips Intera MR scanner with 8‐channel head coil (Philips Medical System, Best, The Netherlands) was used for MRI acquisition across all centers. The following brain MRI sequences were acquired from all subjects during a single session: (a) 3DT1‐weighted turbo field echo (repetition/echo time = 25/4.6 ms, echo train length = 1, flip angle = 30°, matrix size = 256 × 256, field‐of‐view = 230 × 230 mm^2^, 220 contiguous, axial slices with voxel size = 1 × 1 × 1 mm) (b) dual‐echo turbo spin echo yielding proton density (PD) and T2‐weighted images (repetition/echo time = 2599/16.80 ms, echo train length = 6, flip angle = 90°, matrix size = 256 × 256, field‐of‐view = 240 × 240 mm^2^, 44 axial 3‐mm‐thick slices). For all sequences, slices were positioned to run parallel to a line joining the most infero‐anterior and infero‐posterior margins of the corpus callosum.

### MRI data analysis

T2‐hyperintense lesion volumes (LV) were measured on PD images, using a local thresholding semi‐automated segmentation technique (Jim 8, Xinapse Systems, Colchester, United Kingdom). Normalized brain (NBV), white matter (NWMV), gray matter (NGMV) and cortical GM (NcGMV) volumes were measured on lesion‐filled^18^ 3DT1‐weighted images using SIENAx software. Automated segmentation of thalamus, caudate, putamen, pallidum, hippocampus, amygdala, and nucleus accumbens was performed on lesion‐filled^18^ 3DT1‐weighted images with FMRIB's Integrated Registration and Segmentation Tool (FIRST; http://fsl.fmrib.ox.ac.uk/fsl/fslwiki/FIRST) software.^19^ The volume of these structures was multiplied by the head normalization factor derived from SIENAx. Given the symmetry of right and left deep GM nuclei, corresponding volumes were averaged across hemispheres before statistical analysis.^20^ Except for T2 LV, all MRI variables were converted to age‐ and sex‐normalized *z‐*scores, to obtain a standardized measure of deviation from the age‐ and sex‐specific expected reference value, based on our HC cohort.

### Statistical analysis

Between‐group comparisons of demographic and clinical parameters were performed using age‐ and sex‐adjusted linear regression models or non‐parametric tests, as appropriate (normal distribution was assessed by visual inspection and Kolmogorov–Smirnov test). Multinomial regression models were applied to our original cohort to assign a cognitive phenotype to newly enrolled patients. In detail, we used a multinomial regression model to assess the contribution of z‐scores from each cognitive test in predicting cognitive phenotypes. We assigned cognitive phenotypes to our newly enrolled patients based on the membership probabilities estimated directly from the model. Then, multinomial regression models were used to estimate the relationship between cognitive phenotypes and disease duration (calculated as the time from disease onset to study enrolment), considering the effect of age at disease onset. To examine the nature of the statistical interaction of age at disease onset and disease duration, we compared the probability of belonging to a specific cognitive phenotype at prespecified disease duration timepoints (1, 10, and 20 years) in POMS vs AOMS and in AOMS vs EOMS.

By using the binomial regression model, we also estimated and compared among the same groups, the risk of having a *z*‐score <−1.645 (i.e., the healthy population 5th percentile) in cortical and subcortical GM volume as well as the median of lesion volume distribution at the same disease duration time points. Given the particular relevance of thalamus and hippocampus in MS pathology, we analyzed these structures separately, while grouping caudate, pallidum, putamen, amygdala, and accumbens under “deep GM.” Statistical significance was corrected for multiple comparisons (false discovery rate method), and threshold for significance was set at corrected *P* < 0.05. Statistical analysis was performed by using R software 4.2.2.

## Results

### Demographic, clinical, neuropsychological, and MRI measures

Table 1 summarizes the main demographic clinical, neuropsychological, and MRI features of study subjects as a whole and grouped according to age at disease onset. Compared to HC, MS patients did not differ in age, sex, and education. Lower thalamus, hippocampus, and deep gray matter volumes were observed in MS patients compared to HC.

**Table 1:** Demographic and clinical features of HC and MS patients, as a whole and grouped by age at disease onset.

	HC	MS patients	HC *vs* all MS patients *P* values	AOMS patients	POMS patients	AOMS vs. POMS *P* values	EOMS patients	AOMS vs. EOMS *P* values
*N*	238	1262	‐	1066	140		56	‐
Mean age (SD) [years]	41.2 (10.1)	40.8 (11.4)	0.68	41.3 (10.5)	30.4 (10.5)	<0.001	58.5 (5.3)	<0.001
Female/male	157/81	818/444	0.64	693/373	94/46	0.63	34/22	0.69
Education (SD) [years]	12.5 (3.4)	12.4 (3.5)	0.38	12.2 (3.7)	13.0 (3.1)	0.08	11.1 (4.3)	0.002
Clinical phenotype (RR/SP/PP)	‐	1075/119/68	‐	913/96/57	127/12/1	0.58	35/11/10	<0.001
Median EDSS (range)	‐	2.0 (0.0–8.5)	‐	2.0 (0.0–8.5)	2.0 (0.0–8.0)	0.89	3.0 (0.0–6.5)	0.46
Mean age at disease onset (SD) [years]	‐	30.1 (10.1)	‐	30.9 (8.0)	15.2 (2.9)	<0.001	53.3 (3.3)	<0.001
Mean disease duration (SD) [years]	‐	10.7 (9.2)	‐	10.4 (8.9)	15.1 (10.6)	<0.001	5.2 (4.0)	<0.001
Cognitive phenotypes^a^	‐	254/373/237187/211	‐	218/310/193/163/182	32/42/30/17/19	0.67	4/21/14/7/10	0.25
Median T2 lesion volume (IQR) [mL]	‐	4.9 (1.9, 12.1)	‐	4.4 (2.4, 11.0)	4.8 (1.6, 12.3)	0.89	6.2 (3.5, 12.1)	0.35
Normalized cortical gray matter volume (SD) [mL]	686 (49)	649 (76)	0.13	647 (71)	688 (89)	0.99	590 (67)	0.02
Thalamus volume (SD) [mL]	10.4 (0.8)	9.5 (1.2)	<0.001	9.6 (1.5)	9.9 (1.4)	0.15	9.4 (1.2)	0.84
Hippocampus volume (SD) [mL]	5.6 (0.9)	5.3 (1.0)	0.05	5.4 (1.0)	4.9 (0.7)	0.67	5.6 (1.1)	0.41
Deep gray matter (SD) [mL]	16.2 (1.9)	15.2 (2.1)	<0.001	15.3 (2.2)	15.0 (2.0)	0.39	14.5 (1.8)	0.14

AOMS, adult‐onset MS; EDSS, Expanded Disability Status Scale; EOMS, elderly‐onset MS; HC, healthy controls; IQR, interquartile range; MS, multiple sclerosis; POMS, pediatric‐onset MS; PP, primary progressive; RR, relapsing remitting; SD, standard deviation; SP, secondary progressive.

^a^ Cognitive phenotype distribution is reported in the following order: “preserved‐cognition”/“mild verbal‐memory/sematic fluency”/“mild multi‐domain”/“severe executive/attention”/“severe multi‐domain.”

A similar proportion of females and males was observed in POMS, AOMS, and EOMS. POMS were younger and had longer disease duration compared to AOMS; EOMS were older, had shorter disease duration, and had lower education levels compared to AOMS. No differences in distribution of clinical phenotypes were observed between POMS and AOMS; however, a higher proportion of secondary, and primary progressive MS patients was observed in EOMS compared to AOMS. No significant differences were found in terms of clinical disability and cognitive phenotype distribution among groups. Except for a lower NcGMV observed in EOMS compared to AOMS, no significant differences in MRI measures were found among groups.

The multinomial regression model predicted the different cognitive phenotypes with an overall accuracy of 96% with a McFadden's R‐squared of 0.47.

### Relationship between cognitive phenotypes disease duration and age of onset

**Figure 1:**
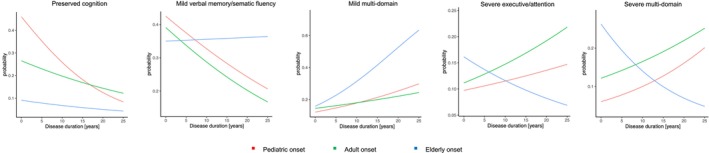
Cognitive phenotypes over increasing disease duration. Summarizes predicted probabilities to belong to each phenotype over increasing disease duration in patients grouped according to the age at disease onset. Pediatric onset = red, adult onset = green, elderly onset = blue.

Table 2 and Fig. 1 summarize the relationship between cognitive phenotypes and disease duration according to the age at onset. In POMS and AOMS, the probability of belonging to the “preserved‐cognition” or “mild verbal memory/semantic fluency” phenotypes decreased with longer disease duration; while the probability of belonging to the “mild multi‐domain” and “severe multi‐domain” increased. For AOMS, the probability of belonging to “severe executive/attention” phenotype increased with longer disease duration, but this was not observed in POMS patients. Overall, no significant differences were observed between POMS and AOMS in the trajectories of probability of belonging to each specific cognitive phenotype over the disease duration.

**Table 2:** Associations between disease duration and the probability to belong to each phenotype, to overcome the median value of T2‐lesion volume distribution and to experience regional gray matter atrophy, according to the age at disease onset and significance of interaction term (pediatric onset*disease duration and elderly onset*disease duration).

	Association with disease duration in AOMS patients		Association with disease duration in POMS patients		Difference in association POMS vs. AOMS patients	Association with disease duration in EOMS patients		Difference in association EOMS vs. AOMS patients
	Beta	*P* values	Beta	*P* values	*P* values	Beta	*P* values	*P* values
Cognitive phenotypes								
Preserved‐cognition	−0.04 [−0.06, −0.02]	<0.001	−0.04 [−0.08, −0.01]	0.006	0.98	−0.24 [−0.49, −0.07]	0.02	0.05
Mild verbal memory/semantic fluency	−0.05 [−0.06, −0.03]	<0.001	−0.03 [−0.06, −0.01]	0.006	0.37	−0.02 [−0.12, 0.07]	0.67	0.55
Mild multi‐domain	0.03 [0.01, 0.04]	<0.001	0.04 [0.01, 0.06]	<0.001	0.35	0.10 [0.00, 0.20]	0.03	0.04
Severe executive‐attention	0.03 [0.01, 0.05]	<0.001	0.03 [−0.02, 0.04]	0.38	0.17	−0.05 [−0.14, 0.09]	0.82	0.79
Severe multi‐domain	0.03 [0.02, 0.05]	<0.001	0.02 [0.00, 0.05]	0.03	0.76	−0.13 [−0–36, 0.05]	0.46	0.25
MRI measures								
T2 lesion volumes	0.07 [0.04, 0.09]	<0.001	0.06 [0.03, 0.11]	<0.001	0.76	−0.08 [−0.24, 0.07]	0.29	0.06
Normalized cortical gray matter volume	0.05 [0.03, 0.08]	<0.001	0.04 [0.01, 0.08]	0.007	0.61	0.13 [0.03, 0.22]	0.006	0.04
Thalamus volume	0.07 [0.05, 0.10]	<0.001	0.05 [0.02, 0.08]	0.002	0.03	0.11 [0.02, 0.19]	0.01	0.71
Hippocampus volume	0.02 [0.00, 0.05]	<0.001	0.04 [0.01, 0.07]	0.006	0.26	0.32 [0.15, 0.56]	0.005	0.04
Deep gray matter volume	0.07 [0.05, 0.10]	<0.001	0.08 [0.05, 0.12]	<0.001	0.35	0.15 [0.05, 0.24]	0.002	0.12

AOMS, adult‐onset MS; EOMS, elderly‐onset MS; MS, multiple sclerosis; POMS, pediatric‐onset MS.

In EOMS, the probability of belonging to the “preserved‐cognition” phenotype decreased, while the probability of belonging to the “mild multi‐domain” phenotype increased with longer disease duration. The probability of belonging to the remaining phenotypes did not show significant associations with disease duration. With longer disease duration, EOMS patients, compared to AOMS, showed a lower probability of belonging to the “preserved‐cognition” phenotype and a higher of belonging to the “mild multi‐domain” phenotype.

At disease duration 1 year, POMS showed a higher probability of belonging to the “preserved‐cognition” and a lower probability of belonging to the “severe multi‐domain” phenotype, compared to AOMS. Compared to AOMS, EOMS showed a lower probability of belonging to the “preserved‐cognition” phenotype and a higher probability of belonging to the “severe multi‐domain” phenotype. No significant differences were observed among groups for the remaining phenotypes.

At disease duration 10 years, no differences in the probability of belonging to any phenotype were observed between POMS and AOMS. However, compared to AOMS, EOMS patients showed a lower probability of belonging to the “preserved‐cognition” phenotype.

At disease duration 20 years, no differences in the probabilities of belonging to any phenotype were observed between POMS and AOMS. EOMS showed a higher probability of belonging to the “mild verbal‐memory/semantic‐fluency” and “mild multi‐domain” and a lower probability of belonging to the “preserved‐cognition” and “severe executive‐attention,” compared to AOMS. Table 3 summarizes between‐group comparisons.

**Table 3:** Group comparisons of probability to belong to each cognitive phenotype at 1, 10, and 20 years of disease duration.

Cognitive phenotypes	Disease duration 1 year *ß* [95% CI]	*P* values (vs. AOMS)	Disease duration 10 years *ß* [95% CI]	*P* values (vs. AOMS)	Disease duration 20 years *ß* [95% CI]	*P* values (vs. AOMS)
Preserved‐cognition						
AOMS	0.25 [0.21, 0.30]		0.20 [0.18, 0.23]		0.14 [0.11, 0.18]	
POMS	0.42 [0.25, 0.59]	0.05	0.26 [0.18, 0.35]	0.18	0.13 [0.04, 0.21]	0.68
EOMS	0.09 [0.00, 0.19]	0.01	0.07 [0.00, 0.16]	0.03	0.04 [0.00, 0.18]	0.05
Mild verbal memory/semantic fluency						
AOMS	0.38 [0.33, 0.42]		0.29 [0.26, 0.32]		0.20 [0.16, 0.24]	
POMS	0.36 [0.20, 0.52]	0.84	0.34 [0.25, 0.44]	0.32	0.26 [0.16, 0.36]	0.28
EOMS	0.34 [0.14, 0.54]	0.90	0.36 [0.14, 0.58]	0.54	0.43 [0.12, 0.74]	0.05
Mild multi‐domain						
AOMS	0.14 [0.11, 0.17]		0.18 [0.16, 0.21]		0.23 [0.19, 0.27]	
POMS	0.09 [0.02, 0.17]	0.27	0.18 [0.10, 0.26]	0.87	0.28 [0.19, 0.37]	0.28
EOMS	0.16 [0.02, 0.32]	0.71	0.31 [0.11, 0.52]	0.21	0.54 [0.29, 0.80]	0.03
Severe executive‐attention						
AOMS	0.11 [0.08, 0.14]		0.15 [0.13, 0.18]		0.19 [0.16, 0.24]	
POMS	0.08 [0.01, 0.15]	0.36	0.12 [0.05, 0.18]	0.33	0.28 [0.19, 0.37]	0.28
EOMS	0.15 [0.00, 0.31]	0.57	0.11 [0.00, 0.27]	0.64	0.03 [0.00, 0.20]	0.04
Severe multi‐domain						
AOMS	0.12 [0.09, 0.15]		0.17 [0.14, 0.19]		0.23 [0.19, 0.26]	
POMS	0.05 [0.00, 0.10]	0.01	0.10 [0.04, 0.16]	0.32	0.18 [0.10, 0.26]	0.26
EOMS	0.24 [0.05, 0.44]	0.05	0.14 [0.00, 0.31]	0.73	0.07 [0.00, 0.26]	0.10

AOMS, adult‐onset MS; CI, confidence interval; EOMS, elderly‐onset MS; MS, multiple sclerosis; POMS, pediatric‐onset MS.

### Pattern of atrophy over increasing disease duration according to age at disease onset

**Figure 2:**
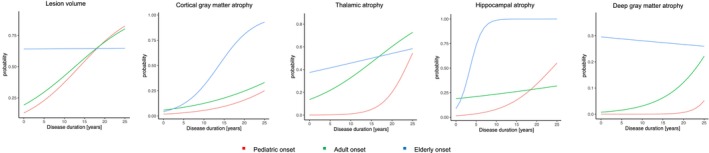
Lesion volume and gray matter atrophy over increasing disease duration. Summarizes predicted probabilities to overcome the median value of lesion volume distribution and to experience gray matter atrophy in cortex, thalamus, hippocampus, and deep gray matter over increasing disease duration in patients grouped according to the age at disease onset. Pediatric onset = red, adult onset = green, elderly onset = blue.

Participants undergoing MRI did not differ from the entire study cohort in terms of demographic, clinical, and neuropsychological variables (data not shown). Table 2 and Fig. 2 summarize the relationship between GM atrophy pattern and disease duration according to the age at onset. All three patient groups (POMS, AOMS, and EOMS) showed an increasing probability of experiencing cortical GM, thalamic, hippocampal, and deep GM atrophy with longer disease duration. A similar trend was observed in the probability of exceeding the median lesion volume distribution in both POMS and AOMS, but not in EOMS. Compared to AOMS, POMS only showed a lower probability of experiencing thalamic atrophy with longer disease duration, while EOMS showed higher probability of experiencing atrophy in cortical GM and hippocampus.

At disease duration 1 year, POMS showed a lower probability of experiencing thalamic atrophy, compared to AOMS. EOMS showed a higher probability of exceeding the median value of lesion volume and experiencing thalamic atrophy, compared to AOMS.

At disease duration 10 years, POMS only showed a lower probability of experiencing thalamic atrophy compared to AOMS. EOMS showed a higher probability of experiencing cortical GM and hippocampal atrophy, compared to AOMS.

**Figure 3:**
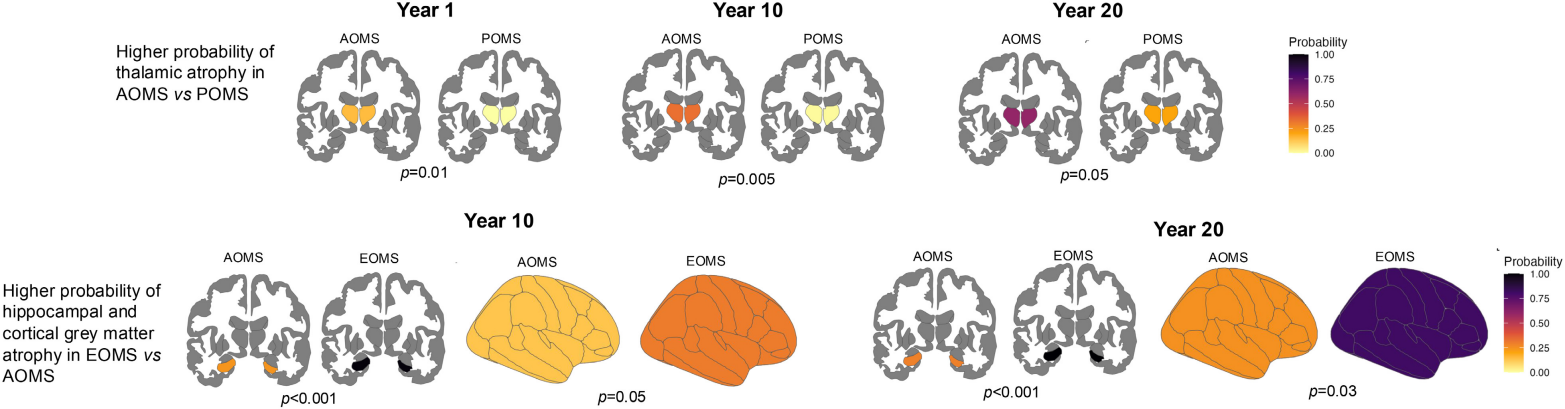
Significant differences between pediatric‐, adult‐ and elderly‐onset. Significant differences among the three groups at 1, 10, and 20 years of disease duration are reported. The probability of experiencing atrophy is shown on a yellow‐black scale. AOMS, adult‐onset multiple sclerosis; EOMS, elderly‐onset multiple sclerosis; POMS, pediatric onset multiple sclerosis.

At disease duration 20 years, POMS showed a lower probability of experiencing thalamic atrophy, compared to AOMS. EOMS showed a higher probability of experiencing cortical GM and hippocampal atrophy, compared to AOMS. Table 4 summarizes between groups comparisons; Fig. 3 summarizes the significant differences in atrophy patterns between POMS and EOMS in comparison to AOMS.

**Table 4:** Group comparisons of probability to overcome the median value of T2‐lesion volume distribution and to experience gray matter atrophy in different regions at 1, 10, and 20 years of disease duration.

MRI measures	Disease duration 1 year; mean probability [95%CI]	*P* values (vs. AOMS)	Disease duration 10 years; mean probability [95% CI]	*P* values (vs. AOMS)	Disease duration 20 years; mean probability [95% CI]	*P* values (vs. AOMS)
T2 lesion volumes						
AOMS	0.15 [0.00, 0.37]		0.43 [0.32, 0.53]		0.70 [0.57, 0.83]	
POMS	0.21 [0.09, 0.33]	0.56	0.37 [0.12, 0.62]	0.65	0.70 [0.36, 1.00]	0.97
EOMS	0.64 [0.31, 0.99]	0.05	0.64 [0.29, 0.98]	0.23	0.65 [0.35, 0.95]	0.90
Normalized cortical gray matter volume						
AOMS	0.06 [0.00, 0.12]		0.13 [0.05, 0.20]		0.25 [0.14, 0.35]	
POMS	0.02 [0.00, 0.08]	0.27	0.06 [0.00, 0.17]	0.89	0.16 [0.00, 0.36]	0.32
EOMS	0.05 [0.00, 0.23]	0.89	0.30 [0.06, 0.54]	0.05	0.81 [0.27, 1.00]	0.03
Thalamus volume						
AOMS	0.15 [0.05, 0.25]		0.33 [0.23, 0.43]		0.60 [0.47, 0.74]	
POMS	0.00 [0.00, 0.01]	0.01	0.01 [0.00, 0.06]	0.005	0.20 [0.00, 0.60]	0.05
EOMS	0.38 [0.15, 0.61]	0.05	0.46 [0.06, 0.85]	0.47	0.54 [0.10, 0.98]	0.88
Hippocampus volume						
AOMS	0.19 [0.08, 0.31]		0.24 [0.15, 0.32]		0.29 [0.18, 0.40]	
POMS	0.07 [0.00, 0.16]	0.08	0.08 [0.00, 0.23]	0.07	0.34 [0.01, 0.67]	0.74
EOMS	0.16 [0.00, 0.46]	0.84	0.99 [0.90, 1.00]	<0.001	1.00 [1.00, 1.00]	<0.001
Deep gray matter volume						
AOMS	0.02 [0.01, 0.04]		0.06 [0.00, 0.11]		0.18 [0.09, 0.28]	
POMS	0.00 [0.00, 0.01]	0.17	0.01 [0.00, 0.05]	0.12	0.12 [0.00, 0.41]	0.61
EOMS	0.14 [0.00, 0.36]	0.37	0.26 [0.08, 0.60]	0.20	0.44 [0.10, 0.78]	0.56

AOMS, adult‐onset MS; CI, confidence interval; EOMS, elderly‐onset MS; MS, multiple sclerosis; POMS, pediatric‐onset MS.

## Discussion

In the present study, we explored cognitive changes specific to POMS and EOMS patients by applying our newly defined cognitive phenotypes.^11^ In detail, we expanded our original cohort and assigned phenotypes to patients by using a probabilistic approach. We described the probability of belonging to each phenotype and experiencing atrophy in different brain regions and high lesion load over the disease course. This approach gave us the opportunity to compare the likelihood of the above‐mentioned events at specific time points in disease duration.

By assessing and comparing the probability of belonging to each cognitive phenotype between POMS and AOMS, we observed that POMS were characterized by better cognitive performance in the earliest phases of disease only. This finding aligns with previous studies showing greater, yet early‐stage‐limited, cognitive resilience in pediatric MS patients compared to their adult counterparts.^21^, ^22^ In detail, pediatric MS patients have been proven to perform significantly better than adults with MS in information processing speed and verbal memory.^23^ Furthermore, it has been observed that individuals with POMS, compared to those with AOMS, perform better on the Symbol Digit Modalities Test in the early stages of the disease, but subsequently experience a greater degree of decline over time than the AOMS group.^22^ Partly in contrast with this last observation, we observed that at 10 and 20 years of disease duration POMS paralleled AOMS in their trajectories, thus demonstrating a similar rate of cognitive decline, later during the disease course. However, we cannot exclude that further extending the disease duration we could find a higher prevalence of severe cognitive phenotypes, and thus a faster rate of cognitive decline, in POMS compared to AOMS.

Analyzing the pattern of GM matter atrophy, we observed that compared to AOMS, POMS patients showed lower probability of developing thalamic atrophy at all the three time points examined. Reduced thalamic volume in pediatric MS patients compared to age‐matched healthy controls has been described.^24^, ^25^, ^26^ However, longitudinal studies within this cohort remain limited, preventing a clear understanding of the extent to which thalamic volume loss can be attributed to impaired GM maturation.^26^, ^27^ Furthermore, the variety of pathogenetic mechanisms underlying thalamic damage^25^ further complicates our understanding of their dynamics. Considering the higher myelin repair capabilities of pediatric MS patients,^28^, ^29^ it could be speculated that remyelination within white matter lesions could mitigate the extent of thalamic damage caused by retrograde Wallerian degeneration at least in the earliest phases of disease.

From a broader perspective, we can speculate that the relatively preserved thalamic volume in POMS compared to AOMS may explain the higher proportion of patients with “preserved cognition” in the early stages of the disease. However, as damage to this structure advances (even in POMS), it may gradually lose its ability to compensate for damage in other areas, which could be progressing at an equal or faster rate than in AOMS, ultimately resulting in similar cognitive profiles. Since we previously demonstrated that “preserved‐cognition” phenotype is characterized by thalamic volume loss in MS patients compared to HC,^11^ there is likely to be a threshold effect of thalamic damage in determining significant cognitive changes.

Analyzing the pattern of cognitive phenotypes in EOMS, it is noteworthy that although the overall likelihood of experiencing a cognitive deficit is higher compared to AOMS, the probability of belonging to “severe multi‐domain” is higher at disease onset only. Furthermore, after 20 years of disease, EOMS patients, compared to AOMS, exhibit higher probability of belonging to “mild verbal‐memory/semantic‐fluency” and “mild multi‐domain” and lower probability of belonging to “severe executive‐attention” phenotype.

The pattern of MRI abnormalities observed is consistent with the pattern of cognitive changes observed, as EOMS patients, compared to AOMS, have a higher probability of experiencing hippocampal and cortical GM atrophy over the entire disease course, which are substrates of the “mild verbal‐memory/semantic‐fluency” and “mild multi‐domain” phenotypes, respectively.^11^ Furthermore, the absence of an increase in lesion volume can justify the lower probability of belonging to the “severe executive‐attention” phenotype later in the disease course, as observed in EOMS compared to AOMS. Overall, these findings suggest that although the MS effect on the aging brain is likely to lead to cognitive decline, its severity is more influenced by brain or cognitive reserve than by disease duration, as demonstrated by the higher probability of belonging to milder phenotype also later during the disease course.

As confirmed by the absence of lesion load accrual over increasing disease duration, in older patients, inflammatory processes are likely to be unbalanced toward chronic rather than acute neuroinflammation. Interestingly, this chronic low‐grade inflammation, a phenomenon known as “inflammaging” observed in EOMS,^30^ could accelerate damage and volume loss in those regions more susceptible to aging‐related neurodegeneration such as the hippocampus and cerebral cortex.

Indeed, changes in the neuro‐immune profile, including microglial sensitization, appear to be characteristic of the normal aging process, leading to an enhanced and intensified neuroinflammatory response in the aging brain following an immune challenge.^2^, ^31^, ^32^ Such responses may culminate in prolonged increases in pro‐inflammatory cytokines, particularly within the hippocampus.^33^ This, in turn, can result in impaired neural plasticity, volume reduction, and associated cognitive deficits.^32^ Additionally, increased accumulation of activated memory B cells and plasma cells within the meninges and meningeal tertiary follicle‐like structures has been observed in both aging individuals and progressive MS patients.^34^, ^35^, ^36^ This pathological substrate may explain the higher likelihood of experiencing cortical GM atrophy in EOMS compared to AOMS. Indeed, evidence suggests that cortical damage typically occurs in the later stages of the disease when compartmentalized chronic neuroinflammation predominates.^35^

A comprehensive analysis of our findings suggests that more similarities than differences can be observed between POMS and AOMS, while MS onset during aging is likely to result in a distinct cognitive profile and pattern of MRI changes. In detail, the cognitive and MRI changes observed in POMS compared to AOMS may indicate that the greater resilience in POMS against disease‐related damage is limited to the earliest stages of the disease. Early in the disease course, POMS patients are more likely to belong to the “preserved‐cognition” phenotype; however, as the disease progresses, the probability of belonging to each phenotype becomes similar between POMS and AOMS, likely due to the depletion of brain compensatory mechanisms. Conversely, the effect of MS on the aging brain can have a wide range of severity at disease onset, depending on individual cognitive and brain reserve, but appears to have a slower evolution over increasing disease duration, with changes primarily attributable to chronic neuroinflammatory processes that accelerate brain aging.

This study is not without limitations. First, the relatively small number of patients belonging to POMS and EOMS groups, considering the rarity of both pediatric and elderly onset patients. Second, the lack of longitudinal data, which prevented us from confirming the validity of our predicted trajectories. Finally, the limited availability of MRI sequences did not allow us to analyze more specific substrates for chronic neuroinflammation like chronic active lesions or subpial demyelination.

In conclusion our data add to previous knowledge in the field by demonstrating the existence of specific cognitive phenotype distributions and MRI changes over the disease duration according to the age at disease onset. These findings underscore the importance of considering different cognitive rehabilitation strategies according to the age at disease onset. Regarding disease‐modifying treatments, considering the underlying MRI patterns of abnormalities identified according to the age at disease onset, we can speculate that while POMS can benefit more from treatments able to promptly stop acute neuroinflammation and enhance remyelination, EOMS are likely to require treatments that target chronic compartmentalized neuroinflammation and neurodegeneration.

## Author Contributions

E. De Meo contributed to study concept, drafting/revising the manuscript, data collection and analysis. E. Portaccio and M.P. Amato contributed to drafting/revising the manuscript, data collection and analysis. R. Cortese, L. Ruano, B. Goretti, C. Niccolai, F. Patti, C. Chisari, P. Gallo, P. Grossi, A. Ghezzi, M. Roscio, F. Mattioli, C. Stampatori, M. Simone, R. G. Viterbo, R. Bonacchi, Maria A. Rocca, E. Leveraro, N. De Stefano, M. Filippi, and M. Inglese contributed to patients' enrollment and data collection and analysis.

## Conflicts of Interest

All the authors report no conflict of interest related to the present study.

## Data Availability

Data are available from the corresponding author upon reasonable request.
